# Protein Kinase A Is Involved in Neuropathic Pain by Activating the p38MAPK Pathway to Mediate Spinal Cord Cell Apoptosis

**DOI:** 10.1155/2020/6420425

**Published:** 2020-03-23

**Authors:** Yajun Deng, Liang Yang, Qiqi Xie, Fengbiao Yang, Guoqiang Li, Guangzhi Zhang, Shaoping Li, Zuolong Wu, Jing Wang, Xuewen Kang

**Affiliations:** ^1^Department of Orthopaedics, Lanzhou University Second Hospital, Lanzhou, Gansu Province 730030, China; ^2^Key Laboratory of Orthopaedics Disease of Gansu Province, Lanzhou University Second Hospital, Lanzhou, Gansu Province 730030, China; ^3^The International Cooperation Base of Gansu Province for The Pain Research in Spinal Disorders, Lanzhou, Gansu Province 730030, China; ^4^Department of Neurosurgery, Lanzhou University Second Hospital, Lanzhou, Gansu Province 730030, China; ^5^Institute of Neurology of Gansu Province, Lanzhou University Second Hospital, Lanzhou, Gansu Province 730030, China; ^6^The Department of Osteoporosis, The People's Hospital of Baoan District, Shenzhen, Guangdong Province 518000, China

## Abstract

Neuropathic pain is a serious clinical problem to be solved. This study is aimed at investigating protein kinase A (PKA) expression in neuropathic pain and its possible mechanisms of involvement. A neuropathic pain-related gene expression dataset was downloaded from Gene Expression Omnibus, and differentially expressed genes were screened using the R software. cytoHubba was used to screen for hub genes. A spared nerve injury (SNI) rat model was established, and the paw withdrawal threshold was determined using von Frey filaments. Western blotting and immunofluorescence were used to detect the expression and cellular localization, respectively, of key proteins in the spinal cord. Western blot, ELISA, and TUNEL assays were used to detect cell signal transduction, inflammation, and apoptosis, respectively. *Pka* was identified as a key gene involved in neuropathic pain. After SNI, mechanical allodynia occurred, PKA expression in the spinal cord increased, the p38MAPK pathway was activated, and spinal cord inflammation and apoptosis occurred in rats. PKA colocalized with neurons, astrocytes, and microglia, and apoptotic cells were mainly neurons. Intrathecal injection of a PKA inhibitor not only relieved mechanical hyperalgesia, inflammatory reaction, and apoptosis in SNI rats but also inhibited p38MAPK pathway activation. However, intrathecal injection of a p38MAPK inhibitor attenuated mechanical hyperalgesia, inflammation, and apoptosis, but did not affect PKA expression. In conclusion, PKA is involved in neuropathic pain by activating the p38MAPK pathway to mediate spinal cord cell apoptosis.

## 1. Introduction

Neuropathic pain is a chronic pain state caused by primary damage or dysfunction of the nervous system [1]. Common symptoms include spontaneous burning pain or irritation-induced pain [2]. Neuropathic pain is a severely debilitating state that seriously affects the quality of life of patients [3]. Although numerous in-depth studies have been conducted on neuropathic pain, its pathogenesis has not yet been fully elucidated. Recent evidence suggests that the generation of neuropathic pain is related to changes in pain-related expression of genes such as *COMT*, *TRPV1*, *MC1R*, *GCH1*, and *CACNA2D3* [4]. Studies have shown that herpes simplex virus vector-mediated TNF-*α* receptor expression significantly attenuated neuropathic pain [5, 6]. Thus, studying gene expression is important for elucidating the molecular mechanisms of neuropathic pain and discovering potential therapeutic targets.

With the rapid development of sequencing technologies and bioinformatics, microarray analysis has been widely used in biomedical research and clinical screening of genetic variation [7, 8]. In this study, we downloaded a neuropathic pain-related gene expression dataset from the Gene Expression Omnibus (GEO) database [9], and we identified differentially expressed genes (DEGs) using the R software. The DEGs were subjected to Gene Ontology (GO) term and Kyoto Encyclopedia of Genes and Genomes (KEGG) pathway enrichment analyses. A protein-protein interaction (PPI) network was constructed, and hub genes were selected to explore key genes involved in neuropathic pain after nerve injury.

Based on bioinformatics analysis, we identified protein kinase A (PKA) as a key protein involved in neuropathic pain. PKA is a tetrameric complex composed of two catalytic subunits and two regulatory subunits. It transfers phosphate groups from ATP to serine or threonine residues of a specific protein for phosphorylation, and the phosphorylated proteins can regulate the activity of target proteins [10]. Studies have shown that PKA is closely related to inflammatory pain and bone cancer pain [11, 12]. However, it is unclear whether PKA is involved in neuropathic pain caused by nerve damage.

Mitogen-activated protein kinases (MAPKs) are a class of kinases that mediate cellular responses to external stimuli [13]. p38MAPK is an important signaling molecule in neuropathic pain [14]. Activated p38MAPK plays an important role in the generation and maintenance of neuropathic pain by regulating transcription, protein synthesis, and receptor expression in cells [15, 16]. Persaud et al. [17] found that cAMP-dependent PKA mediates IL-24-induced apoptosis in breast cancer cells by phosphorylating p38MAPK. However, the mechanisms of PKA and p38MAPK in neuropathic pain are unknown.

In neuropathic pain, spinal cord cells can undergo apoptosis [18, 19], which is closely related to the p38 MAPK pathway [20, 21]. Therefore, we hypothesized that PKA is involved in neuropathic pain by activating the p38MAPK pathway to mediate spinal cord cell apoptosis. To test this hypothesis, we established a spared nerve injury (SNI) neuropathic pain rat model, and we observed PKA and p38MAPK expression and spinal cord cell apoptosis and explored the interaction between PKA and p38MAPK. The aim of this study was to investigate PKA expression in neuropathic pain and its possible mechanisms of involvement, so as to provide new insights for elucidating the pathogenesis of neuropathic pain.

## 2. Materials and Methods

### 2.1. mRNA Expression Data

The GSE24982 dataset [22], which is based on the GPL1355 platform (Affymetrix Rat Genome 230 2.0 Array) and includes data of 40 dorsal root ganglia (DRG) samples, including 20 spinal nerve ligation (SNL) DRG samples and 20 sham-operated DRG samples, was downloaded from the GEO (https://www.ncbi.nlm.nih.gov/geo) database. We included ipsilateral DRG samples and excluded samples with large outliers, and finally selected 14 DRG samples (seven SNL samples and seven sham operation samples) for bioinformatics analysis.

### 2.2. Data Preprocessing and DEG Screening

The raw data of the GSE24982 dataset were read using the affy package [23] in R (version 3.6.1, https://www.r-project.org/) for raw data format conversion, missing value padding, and background correction. A violin map was generated in R using the ggplot2 package [24] to visualize the results of the dataset normalization. Differential expression analysis was performed using the limma package [25], and DEGs were identified based on the following criteria: adjusted *P* < 0.05 and ∣log2FC∣ > 1. We used the pheatmap package (https://www.cran.r-project.org/web/packages/pheatmap/index.html) to plot a correlation heat map of the samples and the gplots package (https://www.cran.r-project.org/web/packages/gplots/index.html) to draw a heat map of the DEGs.

### 2.3. GO Term and KEGG Pathway Enrichment Analyses

The GO term and KEGG pathway enrichment analyses of the DEGs were conducted using the clusterProfile package [26]. The enrichplot package (https://www.github.com/GuangchuangYu/enrichplot) was used to visualize the enrichment results. *P* < 0.05 was used as a significant gene enrichment criterion.

### 2.4. PPI Network Construction and Hub Gene Selection

A PPI network was constructed using the NetworkAnalyst (version 3.0, https://www.networkanalyst.ca/) platform [27], with a confidence score cutoff ≥ 900. We also conducted PPI analysis using the STRING database (version 11.0, https://www.string-db.org/) [28]. Cytoscape (version 3.7.1) [29] was used to visualize the PPI network. The Cytoscape plug-in cytoHubba [30] was used to select the top 20 hub genes based on the maximum correlation criterion algorithm.

### 2.5. Hub Gene Differential Expression Display

Heat and volcano maps of the hub genes were drawn using the pheatmap and ggplot2 packages to visualize differential hub gene expression.

### 2.6. Animals

Adult male Sprague-Dawley rats, weighing 200–220 g, were purchased from the Lanzhou Veterinary Research Institute of the Chinese Academy of Agricultural Sciences (Animal Center License No.: SCXK (Gan) 2015-0001). The rats were housed at the Experimental Animal Center of the Second Hospital of Lanzhou University (unit license number: SYXK (Gan) 2018-0003) in a temperature-controlled room at 23 ± 1°C, under a 12 h light/dark cycle, with free access to food and water. All animal protocols were approved by the Animal Ethics Committee of the Second Hospital of Lanzhou University and were conducted in accordance with the guidelines of the International Association for the Study of Pain.

### 2.7. Construction of the Neuropathic Pain Model

A SNI neuropathic pain model was established on the left sciatic nerve of rats using the method described by Decosterd and Woolf [31]. Briefly, rats were anesthetized with sodium pentobarbital (1%; 50 mg/kg; intraperitoneal injection). The skin was disinfected and cut perpendicular to the left hind leg, and muscle tissue was bluntly separated. The sacral and common peroneal nerves were ligated, the sural nerve was preserved, and a nerve of approximately 2 mm was cut at the telecentric end of the ligature to prevent the sheared nerve from healing. The muscles and skin were stitched, and the wounds were disinfected to prevent infection. Rats in the Sham group underwent the same procedure, except that the sciatic nerve was not cut.

### 2.8. Mechanical Hyperalgesia Measurement

Mechanical hyperalgesia in rats was measured by the paw withdrawal threshold (PWT) as described by Chaplan et al. [32]. The rats were placed in a clear plastic cage with a metal mesh bottom and allowed to acclimate for 30 min. The rats were stimulated at the lateral border of the left hind paw with von Frey filaments of varying forces (0.6, 1, 1.4, 2, 4, 6, 8, 10, and 15 g) (North Coast Medical, Morgan Hill, CA, USA). The filaments were bent into an S shape for 5–8 s, and contraction responses, including foot lift and lameness, were observed. We performed three measurements on each rat and recorded the average of the three measurements as the final PWT.

### 2.9. Intrathecal Injection of H89 and SB203580

The PKA inhibitor H89 and the p38 MAPK inhibitor SB203580 (both from Selleck Chemicals, Houston, TX, USA) were injected intrathecally according to the method described by Mestre et al. [33]. After rats were anesthetized, a 25 *μ*l microsyringe was inserted into the subarachnoid space through the L4 and L5 intervertebral space, and the reagent was delivered to the cerebrospinal fluid. A typical appendix reflex (sudden lateral movement of the tail) confirmed that the syringe had been successfully inserted into the subarachnoid space. Rats in the SNI, SNI+H89, and SNI+SB203580 groups were given 2% dimethyl sulfoxide, H89 (5 *μ*g/10 *μ*l), and SB203580 (5 *μ*g/10 *μ*l), respectively, on the 3rd day after surgery, once daily for five days.

### 2.10. Western Blotting

The rats were sacrificed after deep anesthesia with sodium pentobarbital, and the spinal cord tissue of the L4–L6 segment was rapidly separated. Spinal cord tissues were homogenized in a lysis buffer containing protease inhibitor for 30 min. The tissue lysates were centrifuged at 12000 rpm at 4°C for 15 min, and the supernatants were collected. Protein concentrations were measured using the Bradford method [34]. Equal amounts of protein were separated by 10% sodium dodecyl-polyacrylamide gel electrophoresis and transferred to a polyvinylidene fluoride membrane. The membrane was blocked in 5% skim milk powder at room temperature for 1 h and then incubated at 4°C overnight with primary antibodies against the following proteins: PKA (anti-mouse, 1 : 500, Santa Cruz, Dallas, TX, USA), p38 (anti-rabbit, 1 : 1000, Proteintech, Wuhan, China), p-p38 (anti-rabbit, 1 : 1000, Cell Signaling Technology, Danvers, MA, USA), TNF-*α* (anti-rabbit, 1 : 1000, Proteintech, Wuhan, China), IL-1*β* (anti-rabbit, 1 : 1000, Bioss, Beijing, China), caspase3 (anti-rabbit, 1 : 1000, Bioss, Beijing, China), caspase9 (anti-rabbit, 1 : 1000, Bioss, Beijing, China), bax (anti-rabbit, 1 : 2000, Proteintech, Wuhan, China), bcl-2 (anti-rabbit, 1 : 1000, Proteintech, Wuhan, China), and *β*-actin (anti-mouse, 1 : 1000, Beyotime, Shanghai, China). The membrane was washed three times with Tris-buffered saline-Tween 20 and incubated with horseradish peroxidase-conjugated goat anti-rabbit antibody (1 : 5000; Beyotime, Shanghai, China) or horseradish peroxidase-conjugated goat anti-mouse antibody (1 : 5000; Beyotime, Shanghai, China) at room temperature for 1 h. Immunolabeled protein bands on membranes were detected using an enhanced chemiluminescence kit, and ImageJ (Version: 1.52q, https://www.imagej.en.softonic.com/) software was used to process the grayscale values of protein bands.

### 2.11. Immunohistochemistry

Under deep anesthesia, the rats were perfused with 0.9% saline through the heart and then with 4% paraformaldehyde. L4–L6 segmental spinal cord tissue was collected and fixed in 4% paraformaldehyde at 4°C overnight. Spinal tissue was continuously dehydrated in 20% and 30% sucrose and cut into 10-*μ*m thick sections in a cryostat. The sections were blocked in 10% goat serum at room temperature for 2 h and incubated at 4°C overnight with primary antibodies against the following proteins: PKA (anti-mouse, 1 : 100, Santa Cruz, Dallas, TX, USA), neuronal nuclei (NeuN) (anti-rabbit, 1 : 50, Proteintech, Wuhan, China), glial fibrillary acidic protein (GFAP) (anti-rabbit, 1 : 500, Servicebio, Wuhan, China), and ionized calcium binding adapter molecule 1 (Iba1) (anti-rabbit, 1 : 500, Servicebio, Wuhan, China). Then, the sections were incubated with CY3-labeled goat anti-rabbit (1 : 300, Servicebio, Wuhan, China), 488-labeled goat anti-mouse (1 : 400, Servicebio, Wuhan, China) fluorescent secondary antibody, or a mixture of the two at room temperature for 1 h. The sections were washed three times with PBS and observed under a fluorescence microscope (Olympus, Tokyo, Japan), and images were acquired.

### 2.12. Enzyme-Linked Immunosorbent Assay (ELISA)

L4–L6 segmental spinal cord tissue was grounded, and the slurry was centrifuged at 3000 rpm at 4°C for 10 min. The supernatants were collected as protein samples. The levels of TNF-*α* and IL-1*β* were measured using a TNF-*α* ELISA kit (Thermo Fisher Scientific, Waltham, MA, USA) and an IL-1*β* ELISA kit (MultiSciences, Hangzhou, China), respectively, per the manufacturers' instructions.

### 2.13. TUNEL Detection

Tissue sections were prepared as described above. The sections were incubated at 4°C overnight with primary antibodies against the following proteins: NeuN (anti-rabbit, 1 : 50, Proteintech, Wuhan, China), GFAP (anti-rabbit, 1 : 1000, Servicebio, Wuhan, China), and Iba1 (anti-rabbit, 1 : 500, Servicebio, Wuhan, China). Then, the sections were incubated with CY3-labeled goat anti-rabbit (1 : 300, Servicebio, Wuhan, China) fluorescent secondary antibody at room temperature for 50 min. TUNEL staining was then carried out using an in situ cell death assay kit (Roche, Mannheim, Germany). Nuclei were counterstained using DAPI (Servicebio, Wuhan, China). The sections were observed under a fluorescence microscope (Olympus).

### 2.14. Statistical Analyses

Data were analyzed using the SPSS software (version: 23.0, https://www.ibm.com/cn-zh/analytics/spss-statistics-software) and are expressed as the mean ± standard deviation (SD). Behavioral data were tested by Shapiro-Wilk, and the data of each group obeyed the normal distribution. Then, behavioral data were analyzed by a two-way repeated measures analysis of variance followed by Bonferroni tests for multiple comparisons. The means of the two groups were compared with Student's *t*-test. *P* < 0.05 was considered statistically significant.

## 3. Results

### 3.1. Data Preprocessing and DEG Screening

**Figure 1:**
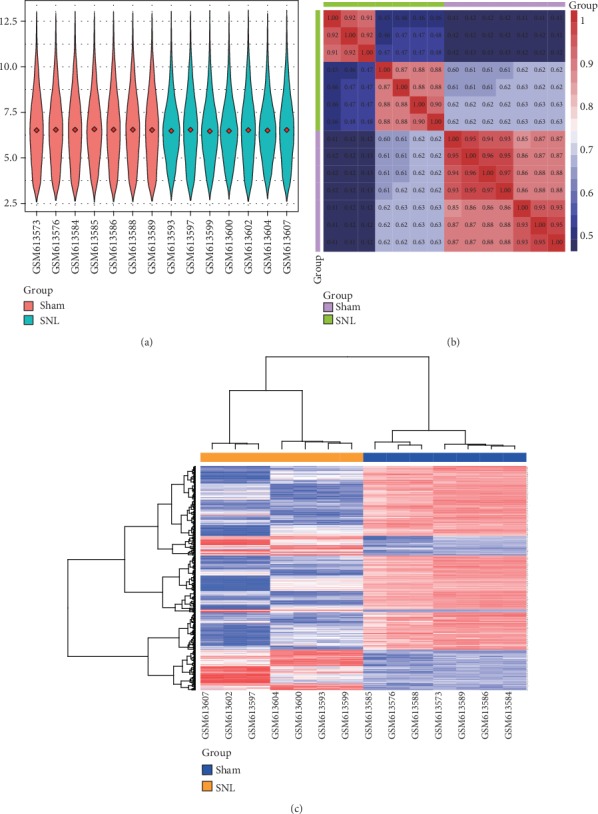
Data preprocessing and differential gene expression analysis. (a) Violin map of the GSE24982 standardized expression data. The red diamond in each sample represents the average gene expression level. (b) Sample correlation heat map of the GSE24982 dataset. Red and blue indicate a strong and weak intersample correlation, respectively. (c) Heat map of the DEGs. Red indicates upregulation; blue represents downregulation.

After standardization of the sample data in the GSE24982 dataset, the average gene expression level was the same in each sample, indicating that consistency was high (Figure 1(a)). A sample correlation heat map showed that the sample source was reliable (Figure 1(b)). In total, 449 DEGs were extracted, and a heat map of these DEGs revealed significant differences in their expression (Figure 1(c)).

### 3.2. GO Term and KEGG Pathway Enrichment Analyses

**Figure 2:**
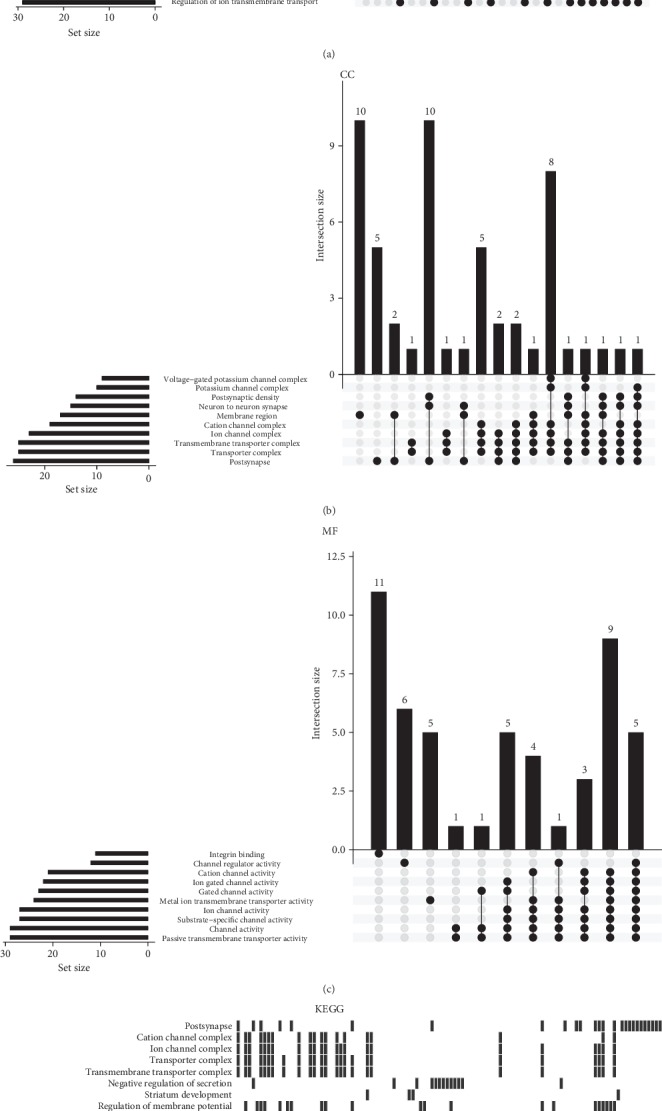
GO term and KEGG pathway enrichment analyses. (a) GO biological process, (b) GO cellular component, and (c) GO molecular function. (d) KEGG analysis.

GO analysis results showed that the DEGs were significantly enriched in the biological processes of multicellular organismal signaling, response to mechanical stimulus, and regulation of ion transmembrane transport (Figure 2(a)), in cellular components related to the ion channel complex and transmembrane transporter complex (Figure 2(b)), and in the molecular functions of substance-specific channel activity and passive transmembrane transporter activity (Figure 2(c)). In KEGG pathway enrichment analysis, enriched pathways were related to leukocyte aggregation, response to mechanical stimulus, and sensory perception of pain pathways (Figure 2(d)).

### 3.3. PPI Network Analysis and Hub Gene Selection

**Figure 3:**
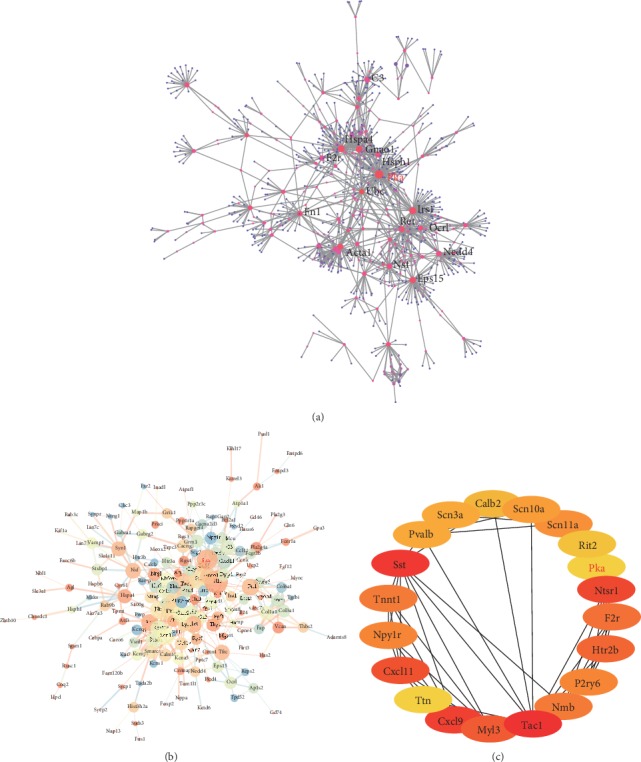
PPI network analysis and identification of hub genes. (a) PPI network based on the NetworkAnalyst database. (b) PPI network based on the STRING database. The node size indicates the clustering coefficient, the node color indicates the representation degree, the line thickness indicates the comprehensive score, and the line color indicates coexpression. (c) Schematic diagram of the hub genes. Red, brown, and yellow indicate a high, low, and intermediate enrichment score, respectively.

PPI network analysis revealed that PKA had relatively large nodes and multiple connections, indicating that it may play an important role in the progression of neuropathic pain (Figures 3(a) and 3(b)). The top 20 hub genes are shown in Figure 3(c). Based on this analysis, we speculated that *Pka* may be a key gene in the progression of neuropathic pain.

### 3.4. Differential Expression of the Hub Genes

**Figure 4:**
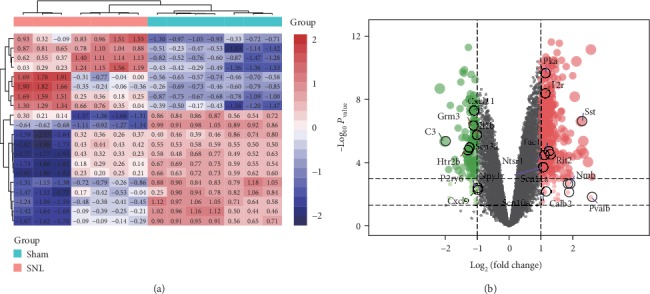
Differential expression of the hub genes. (a) Heat map of the hub genes. Red indicates upregulation; blue indicates downregulation. (b) Volcano map of the hub genes. Red, green, and black dots indicate upregulated, downregulated, and unaltered genes, respectively.

To visualize the differential expression of the hub genes, we generated a heat map (Figure 4(a)) and a volcano plot (Figure 4(b)). It can be seen from Figure 4 that *Pka* was significantly upregulated in SNL samples, suggesting that it may play an important role in the development of neuropathic pain. Thus, we postulated *Pka* as a key gene in neuropathic pain.

### 3.5. SNI Induces Neuropathic Pain, Upregulation of Spinal Cord PKA, and Activation of p38MAPK

**Figure 5:**
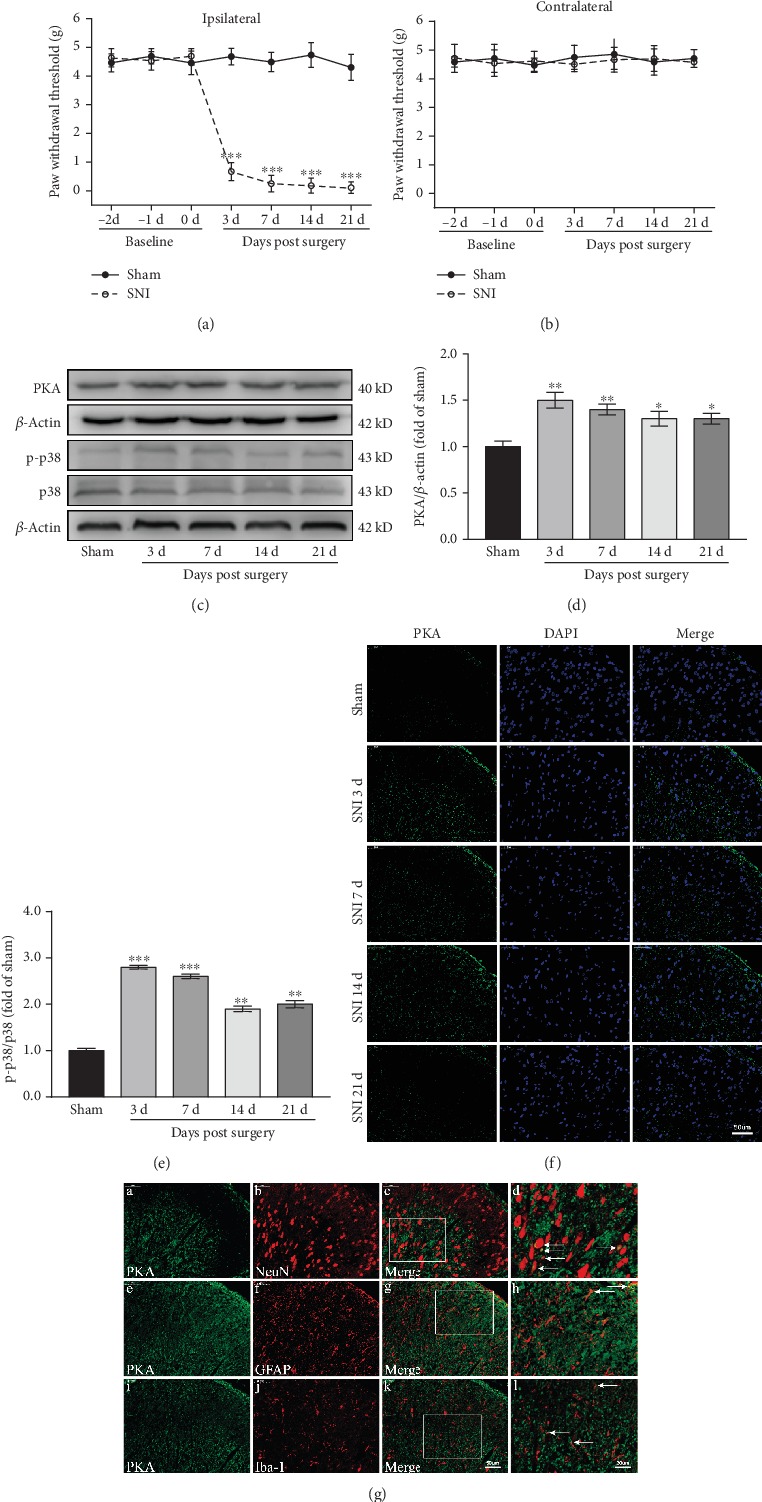
SNI causes neuropathic pain and increases the expression of PKA and p-p38 in the spinal cord. (a, b) SNI ipsilateral PWT (a) and contralateral PWT (b) (*n* = 9). ^∗∗∗^*P* < 0.001 versus Sham group. (c–e) Western blots and quantification of PKA and p-p38 expression in the spinal cord (*n* = 3). ^∗^*P* < 0.05, ^∗∗^*P* < 0.01, ^∗∗∗^*P* < 0.001 versus Sham group. (f) Immunofluorescence images showing PKA expression (green) in the dorsal horn of the spinal cord. (g) Immunofluorescence images showing the distribution of PKA in the cells of the dorsal horn of the spinal cord. PKA is green, NeuN, GFAP, and Iba-1 are red.

As shown in Figure 5(a), there was no significant difference in ipsilateral PWT between the SNI and Sham groups three days before operation (*P* > 0.05). Compared with the Sham group, the ipsilateral PWT in the SNI group was significantly reduced (*F*_(4, 32)_ = 159.56, *P* < 0.001) (Figure 5(a)). There was no significant difference in the contralateral PWT between the two groups (*P* > 0.05) from three days before to 21 days after surgery (Figure 5(b)). These behavioral data indicated that the SNI model was successfully established. Western blotting results showed that PKA expression in the spinal cord increased rapidly and persistently in the SNI group. Compared with the Sham group, PKA increased significantly and reached a peak level on the 3rd day after surgery (*P* < 0.01), which was maintained until the 21st day (*P* < 0.05) (Figures 5(c) and 5(d)). Immunofluorescence results showed that PKA was mainly expressed in the ipsilateral spinal dorsal horn in the SNI group, and the expression trend was consistent with the western blotting results (Figure 5(f)). The levels of p-p38 in the SNI group peaked on the 3rd and 7th days after surgery (*P* < 0.001) and gradually decreased to the 21st day (*P* < 0.01) (Figures 5(c) and 5(e)). These results were consistent with the behavioral changes in rats, suggesting that the PKA and p38 MAPK pathways may be involved in the induction and maintenance of neuropathic pain.

### 3.6. SNI Enhances PKA Expression in Neurons and Glial Cells

We observed the distribution of PKA in the cells of the dorsal horn of the spinal cord by immunofluorescence double labeling. Rat spinal cord tissues were collected on day seven after SNI and double-stained for PKA and Neun, GFAP, or Iba-1. As shown in Figure 5(g), PKA was colocalized with Neun, GFAP, and Iba-1. PKA was mainly expressed in neurons and at a low level in astrocytes and microglia.

### 3.7. SNI Induces Inflammation and Apoptosis in the Spinal Cord

**Figure 6:**
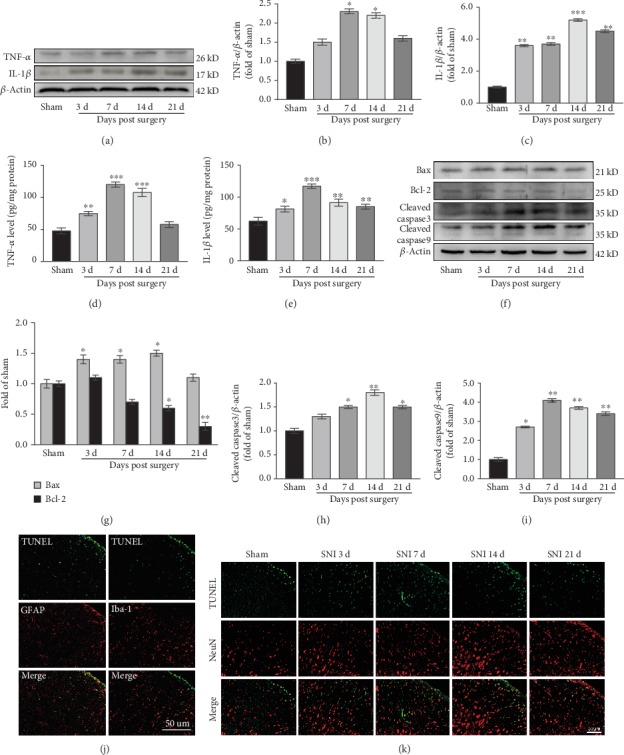
SNI induces inflammation and apoptosis in the spinal cord. (a–c) Western blots and quantification of TNF-*α* and IL-1*β* expression in the spinal cord (*n* = 3). ^∗^*P* < 0.05, ^∗∗^*P* < 0.01, ^∗∗∗^*P* < 0.001 versus Sham group. (d, e) TNF-*α* (d) and IL-1*β* (e) levels in the spinal cord as measured by ELISA (*n* = 3). ^∗^*P* < 0.05, ^∗∗^*P* < 0.01, ^∗∗∗^*P* < 0.001 versus Sham group. (f–i) Western blots and quantification of bax, bcl-2, caspase3, and caspase9 expression in the spinal cord. ^∗^*P* < 0.05, ^∗∗^*P* < 0.01 versus Sham group. (j, k) Double labeling for TUNEL and GFAP, Iba-1, or NeuN. TUNEL-positive cells are mainly colocalized with NeuN (k), a small amount colocalizes with GFAP and Iba-1 (j). TUNEL-positive cells are green, NeuN, GFAP, and Iba-1 are red.

To evaluate the inflammatory reaction of spinal cord tissue after SNI, we determined the expression levels of TNF-*α* and IL-1*β*. Western blotting showed that compared with the Sham group, TNF-*α* levels increased from the 3rd day after SNI, reached a peak on the 7th day (*P* < 0.05) that was maintained until the 14th day (*P* < 0.05), and returned to the levels observed in the Sham group on the 21st day (Figures 6(a) and 6(b)). IL-1*β* expression increased from the 3rd day (*P* < 0.01) and reached a peak on the 14th day (*P* < 0.001) that was maintained until the 21st day (*P* < 0.01) (Figures 6(a) and 6(c)). TNF-*α* and IL-1*β* ELISA results were consistent with the western blotting results (Figures 6(d) and 6(e)). To evaluate apoptosis of spinal cord cells after SNI, we examined the expression of bax, bcl-2, caspase3, and caspase9. The results showed that bax levels increased from the 3rd day after SNI (*P* < 0.05), peaked on the 14th day (*P* < 0.05), and decreased to normal levels on the 21st day (*P* > 0.05) (Figures 6(f) and 6(g)). In contrast, bcl-2 levels gradually declined, with a statistically significant difference on the 14th day (*P* < 0.05), and the lowest levels observed on the 21st day (*P* < 0.01) (Figures 6(f) and 6(g)). The expression of caspase3 gradually increased, reaching a peak on the 14th day (*P* < 0.01) that was maintained to the 21st day (*P* < 0.05) (Figures 6(f) and 6(h)). Like caspase3, caspase9 expression was significantly elevated (*P* < 0.05) on the 3rd day after SNI and reached a peak level on the 7th day (*P* < 0.01) that was maintained until the 21st day (*P* < 0.01) (Figures 6(f) and 6(i)). These data indicated that SNI induces inflammation and apoptosis in the spinal cord.

### 3.8. Neurons and Glial Cells Undergo Apoptosis

To clarify the apoptotic cell types further, we subjected spinal cord tissues to double labeling for TUNEL and GFAP, Iba-1, or Neun. The results showed that TUNEL-positive cells colocalized mainly with neurons (Figure 6(k)), and a small number colocalized with astrocytes and microglia (Figure 6(j)), indicating that spinal cord apoptosis mainly occurred in the neurons. Apoptosis mainly occurred on the 7th and 14th days after SNI (Figure 6(k)), which was consistent with the western blotting results.

### 3.9. PKA and p38MAPK Inhibitors Reduce Neuropathic Pain

**Figure 7:**
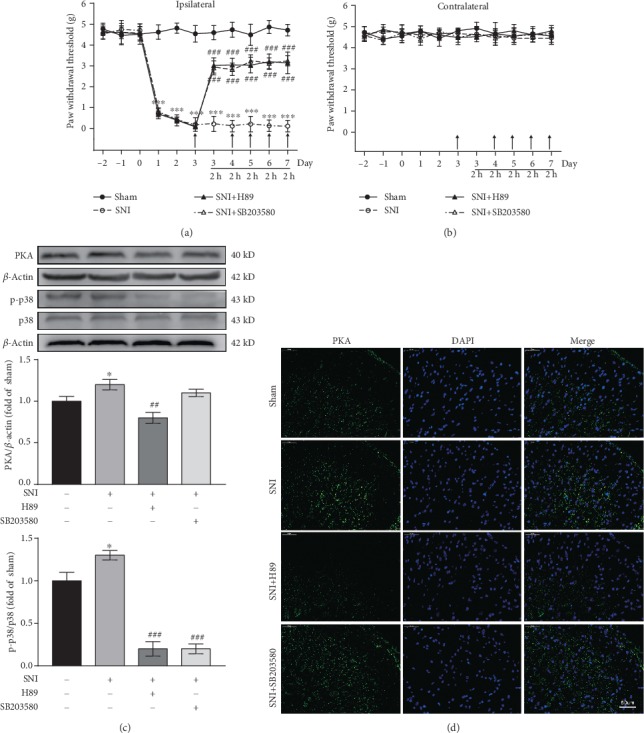
Intrathecal injection of PKA and p38MAPK inhibitors alleviates mechanical hyperalgesia. (a, b) Ipsilateral PWT (a) and contralateral PWT (b) after intrathecal injection of PKA and p38MAPK inhibitors (*n* = 9). Arrows indicate the time points at which inhibitors were administered. PWT was measured 2 h after inhibitor injection. ^∗∗∗^*P* < 0.001 versus Sham group, ###*P* < 0.001 versus SNI group. (c) Western blots and quantification of spinal cord PKA and p-p38 expression (*n* = 3). ^∗^*P* < 0.05 versus Sham group, ##*P* < 0.01, ###*P* < 0.001 versus SNI group. (d) Immunofluorescence images showing PKA expression in the dorsal horn of the spinal cord. PKA is green.

To verify whether the PKA and p38MAPK pathways play key roles in the maintenance of neuropathic pain, PKA and p38MAPK inhibitors were injected continuously for five days from the 3rd day after SNI, and behavioral changes were observed 2 h after injection. As shown in Figure 7(a), the SNI ipsilateral PWT was significantly increased in the SNI+H89 group (*F*_(5, 40)_ = 67.84, *P* < 0.001) and SNI+SB203580 group (*F*_(5, 40)_ = 69.69, *P* < 0.001) when compared with the SNI group. However, there were no significant differences in the SNI contralateral PWT between the SNI+H89 and SNI+SB203580 groups and the SNI group (*P* > 0.05) (Figure 7(b)). These data indicated that PKA and p38MAPK inhibitors can reduce neuropathic pain.

### 3.10. Inhibition of PKA Activation Results in Downregulation of p-p38 Expression

To explore the relationship between PKA and p-p38 further, we examined PKA and p-p38 expression in spinal cord tissues of the rats after injection of PKA and p38MAPK inhibitors. PKA and p-p38 expression was increased in the SNI group when compared with the Sham group (both *P* < 0.05) (Figure 7(c)). Compared with that in the SNI group, PKA activation was inhibited and p-p38 expression significantly decreased in the SNI+H89 group (*P* < 0.01, *P* < 0.001, respectively) (Figure 7(c)). In the SNI+SB203580 group, p38MAPK inhibitor significantly inhibited the activation of p-p38 (*P* < 0.001), but had no effect on PKA (*P* > 0.05) (Figure 7(c)). These data suggested that PKA is upstream of the p38MAPK pathway and may be involved in the maintenance of neuropathic pain by modulating p38MAPK. In addition, immunofluorescence was used to detect PKA expression in each group, and the results were consistent with those of western blotting (Figure 7(d)).

### 3.11. PKA and p38MAPK Inhibitors Reduce Inflammation and Apoptosis

**Figure 8:**
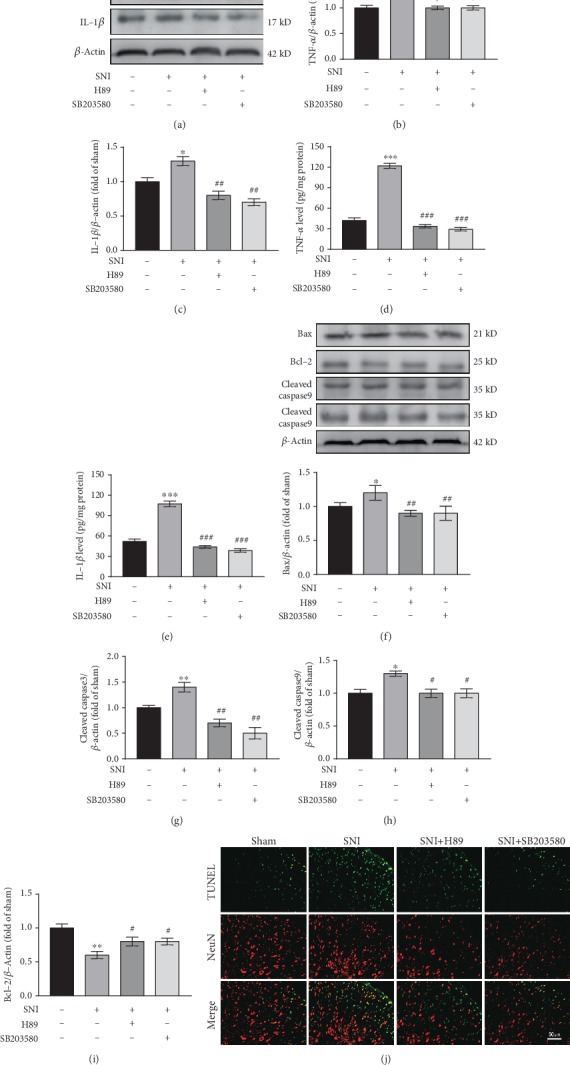
PKA and p38MAPK inhibitors reduce inflammation and apoptosis. (a–c) Western blots and quantification of TNF-*α* and IL-1*β* expression in the spinal cord (*n* = 3). ^∗^*P* < 0.05 versus Sham group, #*P* < 0.05, ##*P* < 0.01 versus SNI group. (d, e) TNF-*α* (d) and IL-1*β* € levels in the spinal cord as measured by ELISA (*n* = 3). ^∗∗∗^*P* < 0.001 versus Sham group, ###*P* < 0.001 versus SNI group. (f–i) Western blots and quantification of bax, bcl-2, caspase3, and caspase9 expression in the spinal cord (*n* = 3). ^∗^*P* < 0.05, ^∗^*P* < 0.01 versus Sham group, #*P* < .05, ##*P* < 0.01 versus SNI group. (j) TUNEL and NeuN double labeling. TUNEL-positive cells are green; NeuN is red.

To validate the roles of the PKA and p38MAPK pathways in neuropathic pain, we observed inflammatory reactions and apoptosis in spinal cord tissues after intrathecal injection of PKA and p38MAPK inhibitors. Western blotting was used to detect changes in inflammatory reaction. The results showed that TNF-*α* and IL-1*β* were significantly increased in the SNI group as compared with the Sham group (both *P* < 0.05) (Figures 8(a)–8(c)). Compared with the levels in the SNI group, TNF-*α* and IL-1*β* expression was decreased in the SNI+H89 group (*P* < 0.05, *P* < 0.01, respectively) and the SNI+SB203580 group (*P* < 0.05, *P* < 0.01, respectively) (Figures 8(a)–8(c)). ELISA results were consistent with those of western blotting (Figures 8(d) and 8(e)). The above data indicated that inhibition of PKA and p38 MAPK can attenuate spinal cord inflammation.

In addition, we used western blotting and TUNEL double labeling to detect apoptosis. Western blotting showed that the expression of bax, caspase3, and caspase9 was higher in the SNI group than in the Sham group (*P* < 0.05, *P* < 0.01, *P* < 0.05, respectively) (Figures 8(f)–8(h)), whereas bcl-2 expression was significantly lower (*P* < 0.01) (Figures 8(f) and 8(i)). Compared with the SNI group, the expression of bax, caspase3, and caspase9 was decreased in the SNI+H89 group (*P* < 0.01, *P* < 0.01, *P* < 0.05) (Figures 8(f)–8(h)), whereas bcl-2 expression was increased (*P* < 0.05) (Figures 8(f) and 8(i)). The expression of bcl-2, bax, caspase3, and caspase9 in the SNI+SB203580 group was consistent with that in the SNI+H89 group (Figures 8(f)–8(i)). As shown in Figure 8(j), TUNEL and NeuN double labeling revealed a significant increase in TUNEL-positive cells in the SNI group as compared with the Sham group. TUNEL-positive cells in the SNI+H89 and SNI+SB203580 groups were lower than those in the SNI group. These results suggested that inhibition of PKA and p38MAPK can attenuate apoptosis.

## 4. Discussion

Neuropathic pain is caused by damage to the nervous system and can be severe [1]. Traditional drug treatments tend to have poor effects; thus, it is important to find new therapeutic targets for neuropathic pain. With the rapid development of molecular biotechnology, the role of pain-related genes in the development of neuropathic pain has become an important research topic. One study has shown that neuropathic pain is often accompanied by changes in the expression of genes involved in the sensory pathway [35]. In-depth analysis of gene expression data by bioinformatics methods might aid in unraveling the complex regulatory relationships between genes and help us find better therapeutic targets for neuropathic pain.

In this study, based on rat neuropathic pain-related mRNA expression data, we identified 449 DEGs in neuropathic pain. Based on PPI network analysis and hub gene identification, we found that *Pka* may play an important role in the occurrence and progression of neuropathic pain. Therefore, we preliminarily speculated that *Pka* may be a key gene in the pathogenesis of neuropathic pain. To verify this speculation, we established a SNI rat model. The mechanical pain threshold of rats was significantly decreased after SNI, which was accompanied by upregulation of PKA in the ipsilateral spinal dorsal horn. PKA was found to be expressed in both neurons and glial cells. This suggested that PKA may be involved in the maintenance of neuropathic pain. After intrathecal injection of a PKA inhibitor in rats, not only was PKA expression suppressed but also the pain response was alleviated. These data suggest that PKA plays a key role in the development of neuropathic pain.

In neuropathic pain, the p38MAPK pathway is activated, which has been confirmed in SNL [36], chronic constriction injury [37], and SNI [38] animal models. The results of the above studies are consistent with our experimental finding that p-p38 expression is upregulated in the spinal cord of SNI rats. When rats were injected intrathecally with p38MAPK inhibitor, p-p38 expression was decreased, which was accompanied by a reduction in the SNI-induced pain response. These data suggest that the p38MAPK pathway is involved in the maintenance of neuropathic pain.

There is an important relationship between PKA and p38MAPK. It has been reported that p38MAPK acts as a downstream mediator of cAMP/PKA signaling and regulates the thermogenic process in brown adipocytes [39]. IL-24 is involved in breast cancer progression through the cAMP-dependent PKA pathway, which regulates the p38MAPK pathway [17], suggesting that the p38MAPK signaling pathway may be the downstream of PKA. To determine the relationship between PKA and p38MAPK in neuropathic pain, we intrathecally injected a PKA inhibitor into rats. We found that p-p38 expression was downregulated with a decrease in PKA, and mechanical pain was alleviated. After injection of a p38 MAPK inhibitor, the decrease in p-p38 did not affect PKA expression, despite the reduction in mechanical pain in the rats. These data indicate that the p38MAPK signaling pathway is the downstream of PKA and is positively regulated by PKA.

Spinal cord cell apoptosis reported is observed during the development of neuropathic pain [18, 19]. Whiteside et al. [40] found that the development of chronic constriction injury-induced neuropathic pain is associated with increased apoptosis in spinal cord cells, which is thought to be the cause of pain. Our data provide new evidence for this in that in the SNI-induced neuropathic pain model, on the 7th and 14th days after surgery, significant apoptosis was observed in the ipsilateral spinal cord dorsal horn tissue, whereas apoptosis was alleviated on the 21st day. Moreover, TUNEL-positive cells mainly colocalized with neurons, and only a few colocalized with astrocytes and microglia, indicating neurons mainly in the spinal cord undergo apoptosis. When neuropathic pain occurs, central sensitization can reduce the pain threshold of dorsal horn neurons and increase the receptive field [41]. Dorsal horn neurons can exhibit pathologically high performance and metabolism, which may be one of the reasons for neuronal apoptosis. Suppressive interneuron apoptosis attenuates the inhibition of nociceptive information transmission, thereby increasing the excitability of nociceptive neurons and producing hyperalgesia [42]. Our data suggest that apoptosis occurs in the early stages of pain induction and maintenance. Thereafter, the process of apoptosis appears to slow down, which may be due to neuroprotective mechanisms of the nervous system itself [43]. SNI induced inflammation in the spinal cord and increased TNF-*α* and IL-1*β* expression. In line herewith, numerous studies have shown that the proinflammatory factors TNF-*α* and IL-1*β* can induce apoptosis [44, 45].

## 5. Conclusions

**Figure 9:**
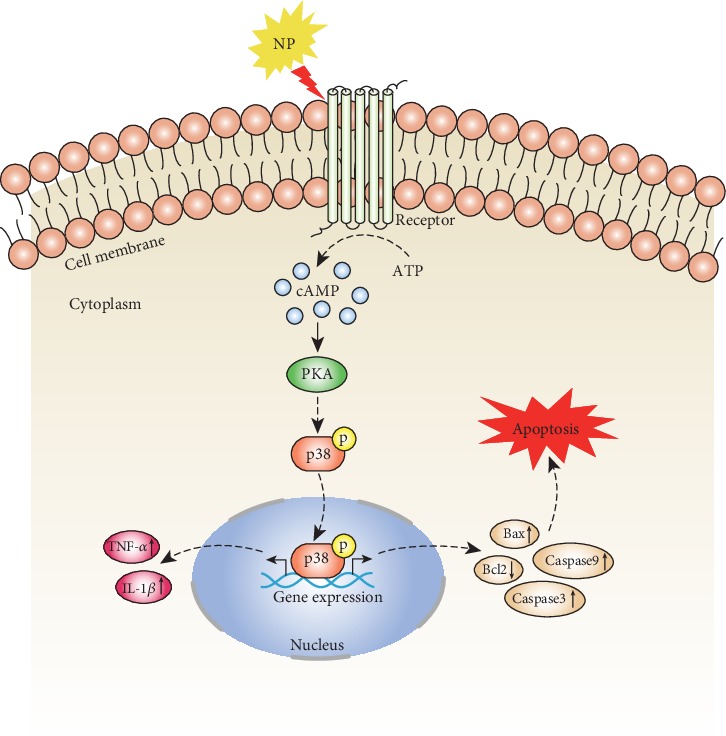
Schematic diagram showing that PKA is involved in neuropathic pain by activating the p38MAPK pathway to mediate spinal cord cell apoptosis.

Our results suggest that SNI induces a significant increase in PKA expression in the spinal cord, and PKA is involved in neuropathic pain by activating the p38MAPK pathway to mediate spinal cord cell apoptosis (Figure 9). This study provides new insights that may aid in the elucidation of the pathogenesis of neuropathic pain.

## Data Availability

The data used to support the findings of this study are available from the corresponding author upon request.
